# Cancer mortality does not differ by antiarrhythmic drug use: A population-based cohort of Finnish men

**DOI:** 10.1038/s41598-018-28541-4

**Published:** 2018-07-09

**Authors:** Kalle J. Kaapu, Lauri Rantaniemi, Kirsi Talala, Kimmo Taari, Teuvo L. J. Tammela, Anssi Auvinen, Teemu J. Murtola

**Affiliations:** 1 University of Tampere, Faculty of Medicine and Life Sciences, Tampere, Finland; 2 Finnish Cancer Registry, Helsinki, Finland; 3 University of Helsinki and Helsinki University Hospital, Department of Urology, Helsinki, Finland; 4 Tampere University Hospital, Department of Urology, Tampere, Finland; 5 University of Tampere, School of Health Sciences, Tampere, Finland

## Abstract

*In-vitro* studies have suggested that the antiarrhythmic drug digoxin might restrain the growth of cancer cells by inhibiting Na+/K+-ATPase. We evaluated the association between cancer mortality and digoxin, sotalol and general antiarrhythmic drug use in a retrospective cohort study. The study population consists of 78,615 men originally identified for the Finnish Randomized Study of Screening for Prostate Cancer. Information on antiarrhythmic drug purchases was collected from the national prescription database. We used the Cox regression method to analyze separately overall cancer mortality and mortality from the most common types of cancer. During the median follow-up of 17.0 years after the baseline 28,936 (36.8%) men died, of these 8,889 due to cancer. 9,023 men (11.5%) had used antiarrhythmic drugs. Overall cancer mortality was elevated among antiarrhythmic drug users compared to non-users (HR 1.43, 95% CI 1.34–1.53). Similar results were observed separately for digoxin and for sotalol. However, the risk associations disappeared in long-term use and were modified by background co-morbidities. All in all, cancer mortality was elevated among antiarrhythmic drug users. This association is probably non-causal as it was related to short-term use and disappeared in long-term use. Our results do not support the anticancer effects of digoxin or any other antiarrhythmic drug.

## Introduction

Various preclinical studies have suggested that the antiarrhythmic drug digoxin may have antineoplastic effects^1–3^. Digoxin may be able to inhibit growth of lung^4–6^, prostate^7,8^ and pancreatic^9^ tumor cell lines and suppress cancer progression. The anticancer effects have been suggested to be due to inhibition of the plasma membrane Na^+^/K^+^-ATPase which increases intracellular concentration of Ca^2+^, eventually causing apoptosis^7,10^. Another proposed mechanism is inhibition of HIF-1alpha, an important regulator of cell growth^8,11^.

Digoxin use might be associated with a decreased risk of prostate cancer^12^, especially among patients under regular PSA-surveillance^13,14^, but is not associated with prostate cancer-specific survival^15–17^. In a British cohort study there was no association between digoxin use and cancer-specific survival from prostate, breast, respiratory or gastrointestinal cancer^18^. Further, digoxin use was not associated with survival among ovarian cancer patients^19^.

Digoxin has estrogenic effects^20^ and has been associated with an increased risk of breast^21^ and uterine cancer but digoxin users may have a better prognosis and a decreased risk of metastases^22–24^. Digoxin use has also been linked to a higher risk of colorectal cancer^25^ but no difference in cancer-specific survival after diagnosis of colorectal cancer was found in a population-based cohort study^26^.

Studies concerning other antiarrhythmic drugs and cancer survival are sparse. The beta-blocker sotalol is both a K^+^-channel blocker and used clinically as an antiarrhythmic drug. Adrenergic activation is essential for cancer and therefore the use of beta-blockers might be beneficial^27^. We have previously shown in a population-based case-control study that sotalol is associated with a lowered prostate cancer risk^28^ but does not associate with survival^17^. Beta-blockers as a group have been linked with prolonged cancer survival^29^.

We estimated the association between use of digoxin, sotalol or other antiarrhythmic drugs and overall cancer mortality and separately lung, colorectal, pancreatic, liver, bladder, renal and CNS cancer mortality in a population-based cohort of Finnish men.

## Results

### Population characteristics

A total of 78,615 men with data from the SII prescription database were included in the study. Of these 9,023 (11.5%) had used at least one antiarrhythmic drug during the follow-up; 6,329 (8.1%) had used digoxin and 2,304 (2.9%) had used sotalol. The median age at baseline was 59 years among the never-users of antiarrhythmic drugs and 63 years among men with any antiarrhythmic drug use during the follow-up.

During the median follow-up of 17.0 years after baseline, 28,936 (36.8%) men died. There were 8,889 cancer deaths altogether, and the most frequent individual cancers were lung cancer (2,384 deaths), colorectal cancer (861 deaths) and pancreatic cancer (782 deaths) (Table 1).

**Table 1:** Population characteristics in the Finnish Randomized Study of Screening for Prostate Cancer.

	Antiarrhythmic drug use			Digoxin use			Sotalol use		
	Never	Ever	P-value	Never	Ever	P-value	Never	Ever	P-value
***Characteristics of Participants***									
Number of participants	69,592	9,023		72,286	6,329		76,311	2,304	
Median Age (IQR)	59 (55–63)	63 (59–67)	0.00	59 (55–63)	63 (59–67)	0.00	59 (55–63)	63 (59–67)	0.00
Median BMI (IQR)	26.3 (24.2–28.7)	27.2 (24.8–30.3)	0.00	26.3 (24.2–28.7)	27.4 (25.1–30.9)	0.00	26.3 (24.2–29.0)	27.2 (25.0–30.2)	0.00
Baseline cancer diagnosis (any)	2,822 (4.1%)	457 (5.1%)	0.00	2,956 (4.1%)	323 (5.1%)	0.00	3,165 (4.1%)	114 (4.9%)	0.06
*Charlson comorbidity index*			0.00			0.00			0.00
0	50,305 (72.3%)	4,703 (52.1%)		52,097 (72.1%)	2,911 (46.0%)		53,653 (70.3%)	1,355 (58.8%)	
1	3,192 (4.6%)	614 (6.8%)		3,322 (4.6%)	484 (7.6%)		3,683 (4.8%)	123 (5.3%)	
2 or greater	16,095 (23.1%)	3,706 (41.1%)		16,867 (23.3%)	2,934 (46.4%)		18,975 (24.9%)	826 (35.9%)	
***Cancer death***									
Overall cancer death	7,873 (11.3%)	1,016 (11.3%)		8,143 (11.3%)	746 (11.8%)		8,622 (11.3%)	267 (11.6%)	
Lung cancer death	2,090 (3.0%)	294 (3.3%)		2,152 (3.0%)	232 (3.7%)		2,320 (3.0%)	64 (2.8%)	
Colorectal cancer death	770 (1.1%)	91 (1.0%)		792 (1.1%)	69 (1.1%)		846 (1.1%)	15 (0.7%)	
Pancreatic cancer death	714 (1.0%)	68 (0.8%)		734 (1.0%)	48 (0.8%)		762 (1.0%)	20 (0.9%)	
Gastric cancer death	316 (0.5%)	27 (0.3%)		321 (0.4%)	22 (0.3%)		336 (0.4%)	7 (0.3%)	
Hepatic cancer	425 (0.6%)	48 (0.5%)		436 (0.6%)	37 (0.6%)		454 (0.6%)	19 (0.8%)	
Renal cancer	251 (0.4%)	35 (0.4%)		259 (0.4%)	27 (0.4%)		277 (0.4%)	9 (0.4%)	
Non-Hodgkin lymphoma	256 (0.4%)	46 (0.5%)		267 (0.4%)	35 (0.6%)		295 (0.4%)	7 (0.3%)	
Bladder cancer	190 (0.3%)	29 (0.3%)		198 (0.3%)	21 (0.3%)		215 (0.3%)	4 (0.2%)	
Central nervous system cancer	191 (0.3%)	17 (0.2%)		198 (0.3%)	10 (0.2%)		203 (0.3%)	5 (0.2%)	
***Prevalence of medication use***									
NSAIDs	54,837 (78.8%)	7,436 (82.5%)	0.00	57,145 (79.1%)	5,128 (81.0%)	0.00	60,311 (79.0%)	1,962 (85.2%)	0.00
Aspirin	10,732 (15.4%)	1,647 (18.3%)	0.00	11,287 (15.6%)	1092 (17.3%)	0.00	11,894 (15.6%)	485 (21.1%)	0.00
Statins	28,014 (40.3%)	4,840 (53.6%)	0.00	29,540 (40.9%)	3,314 (52.4%)	0.00	31,489 (41.3%)	1,374 (59.6%)	0.00
Antidiabetic drugs	13,321 (19.1%)	2,572 (28.5%)	0.00	13,871 (19.2%)	2,022 (31.9%)	0.00	15,274 (20.0%)	619 (26.8%)	0.00
Antihypertensives	44,472 (63.9%)	8,459 (93.7%)	0.00	46,878 (64.9%)	6,053 (95.6%)	0.00	50,731 (66.5%)	2,200 (95.5%)	0.00
Alpha-blockers	18,442 (26.5%)	2,901 (32.2%)	0.00	19,399 (26.8%)	1,944 (30.7%)	0.00	20,554 (26.9%)	789 (34.2%)	0.00

In general, the use of other drugs (NSAIDs, aspirin, statins, antidiabetic drugs, antihypertensive drugs, alpha-blockers and 5-alpha-reductase inhibitors) was more common and the Charlson comorbidity index (CCI) was higher among antiarrhythmic drug users compared to non-users (Table 1).

### Antiarrhythmic drug use and overall cancer mortality

Antiarrhythmic drug use in general was associated with increased cancer mortality in both age-adjusted and multivariable-adjusted analyses (multivariable-adjusted HR 1.43, 95% CI 1.34–1.53,). A similar risk increase was observed for men with digoxin use (HR 1.59, 95% CI 1.47–1.72) and sotalol use (HR 1.16, 95% CI 1.03–1.31) (Table 2). The risk increase attenuated with increasing amount, duration and intensity of drug use but there was no risk decrease even in long-term use (Table 3). Furthermore, the risk elevation tended to decrease also in lagged analysis estimating long-term effects of antiarrhythmic drug use (Table 2).

**Table 2:** Antiarrhythmic drug use and cancer mortality in Finnish Randomized Study of Screening for Prostate Cancer.

Antiarrhythmic drug use	Overall cancer death^a^		Lung cancer death	Colorectal cancer death	Pancreatic cancer death
	Age-adjusted model	Multivarible-adjusted model^b^	Multivarible-adjusted model^c^	Multivarible-adjusted model^c^	Multivarible-adjusted model^c^
	HR (95%CI)	HR (95%CI)	HR (95%CI)	HR (95%CI)	HR (95%CI)
No use	Ref	Ref	Ref	Ref	Ref
Any use	1.40 (1.31–1.50)	1.43 (1.34–1.53)	1.72 (1.52–1.95)	1.38 (1.11–1.73)	1.02 (0.79–1.31)
Lag 3 v	1.24 (1.15–1.34)	1.26 (1.17–1.36)	1.39 (1.20–1.61)	1.36 (1.07–1.74)	0.98 (0.74–1.30)
Lag 5 v	1.21 (1.12–1.31)	1.23 (1.13–1.33)	1.29 (1.10–1.51)	1.42 (1.10–1.82)	0.99 (0.74–1.33)
**Digoxin use**					
No use	Ref	Ref	Ref	Ref	Ref
Any use	1.60 (1.48–1.73)	1.59 (1.47–1.72)	2.10 (1.82–2.41)	1.59 (1.24–2.05)	1.06 (0.79–1.43)
Lag 3 v	1.35 (1.23–1.47)	1.33 (1.21–1.45)	1.59 (1.34–1.88)	1.53 (1.15–2.02)	1.00 (0.72–1.40)
Lag 5 v	1.30 (1.18–1.44)	1.28 (1.16–1.41)	1.49 (1.23–1.79)	1.59 (1.19–2.14)	0.97 (0.67–1.39)
**Sotalol use**					
No use	Ref	Ref	Ref	Ref	Ref
Any use	1.11 (0.98–1.25)	1.16 (1.03–1.31)	1.10 (0.85–1.41)	0.70 (0.42–1.17)	0.99 (0.63–1.54)
Lag 3 v	1.11 (0.98–1.26)	1.16 (1.02–1.32)	1.07 (0.82–1.39)	0.83 (0.51–1.37)	0.98 (0.61–1.57)
Lag 5 v	1.08 (0.95–1.24)	1.14 (0.99–1.30)	0.98 (0.74–1.31)	0.89 (0.54–1.47)	1.06 (0.66–1.69)

^a^Including lung, prostate, colorectal, pancreatic, gastric, liver, renal, non-Hodgkin lymphoma, bladder and central nervous system cancer.

^b^From Cox regression model adjusted for age, screening trial arm and use of cholesterol-lowering, antidiabetic and antihypertensive drugs, aspirin and other NSAIDs, 5alpha-reductase inhibitors, alpha-blockers and cancer diagnose at baseline.

^c^From Cox regression model adjusted for age and use of cholesterol-lowering, antidiabetic and antihypertensive drugs, aspirin and other NSAIDs, 5alpha-reductase inhibitors, alpha-blockers and cancer diagnose at baseline.

**Table 3:** Cancer mortality by amount, duration and intensity of antiarrhythmic drug use in the the Finnish Randomized Study of Screening for Prostate Cancer.

	All antiarrhythmic drugs			Digoxin			Sotalol		
	Overall cancer mortality	Lung cancer mortality	Pancreatic cancer mortality	Overall cancer mortality	Lung cancer mortality	Pancreatic cancer mortality	Overall cancer mortality	Lung cancer mortality	Pancreatic cancer mortality
	HR (95% CI)^a^	HR (95% CI)^a^	HR (95% CI)^a^	HR (95% CI)^a^	HR (95% CI)^a^	HR (95% CI)^a^	HR (95% CI)^a^	HR (95% CI)^a^	HR (95% CI)^a^
Cumulative quantity of medication use^b^									
**DDD tertiles**									
1^st^ tertile	1.85 (1.67–2.05)	2.22 (1.84–2.67)	1.30 (0.87–1.92)	1.97 (1.76–2.21)	2.47 (2.00–3.04)	1.31 (0.83–2.07)	1.18 (0.97–1.44)	1.29 (0.88–1.88)	0.84 (0.38–1.88)
2^nd^ tertile	1.39 (1.25–1.55)	1.88 (1.56–2.28)	0.95 (0.62–1.45)	1.59 (1.39–1.81)	2.03 (1.59–2.58)	1.14 (0.69–1.88)	1.15 (0.93–1.43)	1.17 (0.77–1.76)	1.07 (0.51–2.25)
3^rd^ tertile	1.10 (0.97–1.25)	1.07 (0.83–1.38)	0.84 (0.54–1.32)	1.22 (1.06–1.41)	1.77 (1.39–2.27)	0.78 (0.45–1.35)	1.14 (0.92–1.42)	0.79 (0.47–1.34)	1.07 (0.51–2.26)
Duration of medication use^c^									
**Year tertiles**									
1^st^ tertile	1.72 (1.56–1.89)	2.14 (1.80–2.55)	1.14 (0.78–1.67)	1.84 (1.66–2.05)	2.46 (2.04–2.96)	1.35 (0.90–2.02)	1.32 (1.09–1.60)	1.53 (1.07–2.18)	0.72 (0.30–1.74)
2^nd^ tertile	1.36 (1.22–1.51)	1.72 (1.41–2.09)	0.77 (0.49–1.23)	1.61 (1.41–1.84)	2.18 (1.72–2.76)	0.86 (0.48–1.52)	1.00 (0.81–1.22)	0.88 (0.57–1.35)	0.74 (0.33–1.65)
3^rd^ tertile	1.13 (0.98–1.30)	1.06 (0.79–1.43)	1.17 (0.75–1.82)	1.17 (0.99–1.38)	1.37 (0.99–1.88)	0.86 (0.47–1.56)	1.21 (0.95–1.53)	0.84 (0.48–1.49)	1.75 (0.90–3.38)
Intensity of medication use (DDDs/year)^d^									
**Intensity tertiles**									
1^st^ tertile	1.91 (1.72–2.11)	2.26 (1.87–2.74)	1.25 (0.83–1.89)	2.13 (1.91–2.38)	2.71 (2.22–3.30)	1.30 (0.82–2.05)	1.19 (0.97–1.46)	1.25 (0.85–1.86)	1.18 (0.59–2.36)
2^nd^ tertile	1.42 (1.26–1.59)	1.75 (1.41–2.16)	1.08 (0.71–1.66)	1.49 (1.28–1.74)	1.93 (1.46–2.56)	0.91 (0.49–1.71)	1.22 (0.99–1.50)	1.04 (0.67–1.61)	1.10 (0.52–2.32)
3^rd^ tertile	1.10 (0.98–1.24)	1.30 (1.05–1.61)	0.80 (0.52–1.23)	1.20 (1.06–1.37)	1.68 (1.33–2.11)	0.97 (0.61–1.53)	1.08 (0.87–1.34)	0.99 (0.63–1.56)	0.71 (0.29–1.71)

^a^From Cox regression model adjusted for age, screening trial arm (only for overall cancer mortality) and use of cholesterol-lowering, antidiabetic and antihypertensive drugs, aspirin and other NSAIDs, and 5alpha-reductase inhibitors and alpha-blockers.

^b^Tertile cut-points for cumulative amount of medication use: All antiarrhythmic drugs combined 1^st^ tertile: 1–280 DDD, 2^nd^ tertile: 281–1,400 DDD, 3^rd^ tertile: more than 1,400 DDD; Digoxin 1^st^ tertile: 1–200 DDD, 2^nd^ tertile: 201–960 DDD, 3^rd^ tertile: more than 960 DDD; Sotalol 1^st^ tertile: 1–200 DDD, 2^nd^ tertile: 201–1,230 DDD, 3^rd^ tertile: more than 1,230 DDD.

^c^Tertile cut-points for cumulative duration of medication use: All antiarrhythmic drugs combined 1^st^ tertile: 1–2 years, 2^nd^ tertile: 3–7 years, 3^rd^ tertile: longer than 7 years; Digoxin 1^st^ tertile: 1–2 years, 2^nd^ tertile: 3–6 years, 3^rd^ tertile: longer than 6 years; Sotalol 1^st^ tertile: 1 year, 2^nd^ tertile: 2–5 years, 3^rd^ tertile: longer than 5 years.

^d^Tertile cut-points for intensity of medication use: All antiarrhythmic drugs combined 1^st^ tertile: 1–116 DDDs/year, 2^nd^ tertile: 117–228 DDDs/year, 3^rd^ tertile: more than 229 DDDs/year; Digoxin 1^st^ tertile: 1–100 DDDs/year, 2^nd^ tertile: 101–170 DDDs/year, 3^rd^ tertile: more than 170 DDDs/year; Sotalol 1^st^ tertile: 1–120 DDDs/year, 2^nd^ tertile: 121–285 DDDs/year, 3^rd^ tertile: more than 285 DDDs/year.

### Antiarrhythmic drug use and individual cancers

Both usage of antiarrhythmic drugs in general and usage of digoxin were associated with increased lung cancer mortality (HR 1.72, 95% CI 1.52–1.95 and HR 2.10, 95% CI 1.82–2.41, respectively). This association was not observed for sotalol use (HR 1.10, 95% CI 0.85–1.41) (Table 2). There were similar trends by amount, duration and intensity as with overall cancer mortality (Table 3).

The results for colorectal cancer mortality were rather similar to those for lung cancer mortality; Antiarrhythmic drug use in general and digoxin use both elevated risk of death (HR 1.38, 95% CI 1.11–1.73 and HR 1.59, 95% CI 1.24–2.05). Usage of sotalol was not associated with the risk for colorectal cancer death (HR 0.70, 95% CI 0.42–1.17) (Table 2).

Pancreatic cancer differed from other cancer types since antiarrhythmic drug use had no influence on pancreatic cancer mortality (HR 1.02, 95% CI 0.79–1.31). Identical findings were observed for digoxin use (HR 1.06, 95% CI 0.79–1.43) and for sotalol use (HR 0.99, 95% CI 0.63–1.54).

Furthermore, antiarrhythmic drug use and digoxin use were associated with elevated risk of death due to non-Hodgkin lymphoma and bladder cancer (Table S1).

### Subgroup analysis

**Figure 1:**
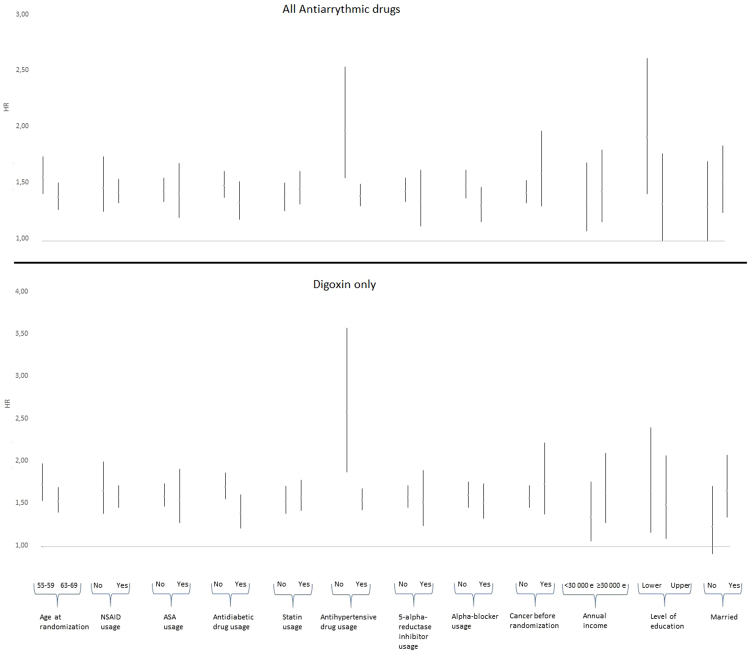
Overall cancer mortality by overall antiarrhythmic drug use and by digoxin use versus non-use stratified by patient characteristics in the the Finnish Randomized Study of Screening for Prostate Cancer.

The overall cancer mortality of antiarrhythmic drug users was increased in all subgroups that we analyzed (Fig. 1). The risk estimates for overall cancer death were most increased among non-users of antihypertensive drugs (p for interaction 0.01). There was a similar risk difference between users and non-users of antihypertensive drugs among digoxin users (p for interaction 0.002). Furthermore, there was an interaction by antidiabetic drug use, the risk being higher among men who were not using antidiabetic drugs (p for interaction 0.01) (Fig. 1).

We used the CCI to stratify the study population by comorbidities. Antiarrhythmic drug use associated with increased cancer mortality among the men with least comorbidities (Charlson index 0: HR 1.37, 95% CI 1.19–1.56). A similar result was observed among men with intermediate comorbidities (Charlson index 1: HR 1.22, 95% CI 0.87–1.71) but the CIs are wider since there were less men in this cohort. There was no association between cancer mortality and antiarrhythmic drug use among men with the most comorbidities (Charlson index 2 or greater: 0.98, 95% CI 0.91–1.06). There was a statistically significant effect modification by CCI (p for interaction < 0.001).

### Sensitivity analysis

To evaluate confounding by indication we estimated the risk association between the indications for antiarrhythmic drug and digoxin use (cardiac insufficiency and arrhythmias) and cancer mortality. 4,199 men had recorded diagnosis of cardiac insufficiency (ICD-10 codes I50) in the HILMO database, while 1,507 men had a diagnosis of arrhythmia (I47 and I49). The increase in overall cancer mortality risk that we observed for cardiac insufficiency (HR 1.19, 95% CI 1.08–1.31) was similar to the risk for antiarrhythmic drug use in general that we observed in our main analysis. However, having a recorded diagnosis of arrhythmia was associated with a lowered risk of cancer death (HR 0.76, 95% CI 0.64–0.90). There was no association between antiarrhythmic drug use and cancer mortality in competing risk analyses. The HR for overall antiarrhythmic drug use was 1.04 (95% CI 0.97–1.12). For digoxin and sotalol users the HRs were 1.01 (95% CI 0.93–1.10) and 1.03 (95% CI 0.91–1.17), respectively.

Both overall antiarrhythmic drug use (HR 1.13, 95% CI 1.05–1.21) and digoxin use (HR 1.13, 95% CI 1.05–1.23) were associated with increased cancer mortality in a sensitivity analysis adjusted by the CCI. However, the risk estimates were lower compared to the main analyses. In this analysis, sotalol use had no effect on cancer mortality (HR 0.98, 95% CI 0.86–1.10). In addition, the CCI was independently associated with an increased risk of cancer death; HR 1.51, 95% CI 1.50–1.52 per increase of one point.

All-cause mortality among antiarrhythmic drug users was increased compared to non-users (HR 2.14, 95% CI 2.07–2.21). Digoxin users had an even greater risk of death (HR 2.52, 95% CI 2.43–2.61), whereas sotalol users had a minor, but nevertheless statistically significant, increase in mortality (HR 1.35, 95% CI 1.27–1.44). Excluding prevalent cancers at baseline from analysis did not modify results (Table S2).

Compared to the users of other antiarrhythmic drugs, digoxin users had an increased risk of cancer death (HR 3.06, 95% CI 2.64–3.54). Sotalol use was not associated with cancer mortality (HR 1.12, 95% CI 0.99–1.32 in a similar sensitivity analysis.

## Discussion

The usage of antiarrhythmic drugs was associated with elevated overall cancer mortality and with increased lung cancer mortality in this retrospective cohort study. Digoxin users had a more prominent increase in risk estimates for cancer death, compared to overall antiarrhythmic drug users. The individual cancer types with increased mortality by digoxin use were lung cancer, colorectal cancer, bladder cancer and non-Hodgkin lymphoma. Usage of sotalol and cancer mortality had no association in the age-adjusted analysis but in the multivariable analysis users had a statistically significant increase in the risk of cancer-specific death.

Digoxin’s mechanism of action differs from other classic antiarrhythmic drugs. Vaughan Williams classification is used to categorize antiarrhythmic agents by mechanism of action. Class I is divided to subclasses Ia, Ib and Ic, all of which are Na^+^-channel blockers. Class II includes beta-blockers (excluding sotalol) and Class III K^+^-channel blockers. Finally, Ca^2+^-channel blockers form class IV and agents with unknown or other mechanisms form class V. Digoxin belongs to the Class V and is a Na^+^/K^+^-ATPase inhibitor. This increases intracellular Na^+^-concentration leading to decreased activity of Na^+^/Ca^2+^-exchanger. Eventually, this cascade results in increased concentration of calcium-ions, which might induce apoptosis^7,10^.

Divergences between users and non-users (confounding by indication) provide a likely explanation for the observed increase in cancer mortality. When we analyzed the association between cardiac insufficiency (the indication) and cancer mortality, a comparable risk elevation was observed. Furthermore, the risk increase tended to disappear with increasing amount, duration and intensity of antiarrhythmic drug use, suggesting that the increased mortality is unlikely to be caused by antiarrhythmic drug use but rather by residual confounding by unmeasured background differences between antiarrhythmic drug uses and non-users. If the drugs did indeed increase the risk, an opposite trend would be presumed.

Digoxin users are likely more fragile than non-users, which may cause non-causal risk differences in epidemiological studies. This explanation was supported by subgroup analyses stratified by the CCI; among men with a low co-morbidity burden, digoxin use was associated with an increased risk of cancer death. However, among men with a high co-morbidity burden, the risk difference disappeared. This confirms that the risk association is modified by background co-morbidities. Further, the CCI was an independent risk factor for cancer death. In the competing risk analyses antiarrhythmic drug use was not associated with cancer mortality, further supporting the notion that use of digoxin or other antiarrhythmic drugs does not affect cancer mortality when non-cancer deaths are taken into account. When compared to users of other antiarrhythmic drugs, digoxin users had an increased cancer mortality. Therefore, the co-morbidity burden may differ even between users of different antiarrhythmic drugs.

Our main results are slightly inconsistent with previously published ones. There are no studies concerning overall cancer mortality and few studies about individual cancer types. *In vitro* studies have suggested that digoxin might have a suppressive effect on lung neoplasms via multiple mechanisms; it has been shown that digoxin hinders tumor progression by inhibiting the activation of an important oncogene Src^4^. Moreover, digoxin decreases the expression of VEGF and NDRG1 through inhibition of HIF-1alpha synthesis^5^ and induces autophagy through the regulation of mTOR and ERK1/2 signaling pathways in non-small cell lung cancer cells^6^. A Swedish study observed that digoxin users had a diminished risk of lung neoplasms (HR 0.55, 95% CI 0.39–0.79) compared to users of organic nitrates^30^. Nonetheless, these chemopreventive features of digoxin did not translate into diminished lung cancer mortality in our large population-based study.

One population-based cohort study regarding colorectal cancer survival has previously been published^26^. The study included 10,357 patients with a colorectal cancer diagnosis and during the median follow-up of 4.8 years 2,724 colorectal cancer–specific deaths occurred. Before model adjustments digoxin use was associated with elevated colorectal cancer–specific mortality (HR 1.25, 95% CI 1.07–1.46), but the association disappeared after adjustment for confounders (HR 1.10, 95% CI 0.91–1.34). In our study, digoxin users had slightly elevated colorectal cancer mortality in the multivariable adjusted analysis. This inconsistency is probably due to differences in adjustment-models. Karasneh *et al*.^26^ were able to adjust the analysis for received radiotherapy, chemotherapy or surgery within 6 months and for comorbidities more comprehensively compared to us.

Interestingly, pancreatic cancer differed from other cancer types. There was no association between pancreatic cancer mortality and digoxin use, whereas there was a statistically significant risk increase for other major cancer types. It has been observed that there is an elevated level of HIF-1alpha expression in pancreatic cancer^31^ and a previous study observed that intraperitoneal digoxin injections significantly reduced pancreatic tumor volume compared to placebo-injections^9^. Furthermore, the same study noticed that digoxin injections decreased the expression of stem cell factor (SCF), a cytokine commonly involved in tumor progression. However, these pathways should be relevant also for other cancer types besides pancreatic cancer. Thus, the differing risk association between digoxin use and pancreatic cancer could be due to other causes.

In contrast to other cancer types, there was no association between sotalol use and death due to lung and colorectal cancer. Sotalol is both a beta-blocker and K^+^-channel blocker and it is possible that these properties may overcome the otherwise increased cancer mortality among antiarrhythmic drug users in these cancer types. Another possibility is that sotalol users had a different distribution of co-morbidities and therefore confounding by indication could have less of an effect. Furthermore, the number of sotalol users was lower compared to digoxin users, resulting in wider confidence intervals and less robust results.

Our study had several strengths. First, we had a large population-based cohort that comprehensively represents the Finnish male population. Additionally, detailed information on antiarrhythmic drug purchases was available, allowing us to calculate the individual amounts and durations of drug use. Consequently, we were able to perform time-dependent regression analyses in order to control for the immortal time bias. We were able to adjust for comorbidities and drugs regularly used along with antiarrhythmic drugs since the information was available from the national registries.

On the other hand, a few limitations should be discussed. There was no information on exact indications of antiarrhythmic drug use even though we were able to separately evaluate the most common indications. We also lacked data on lifestyle habits such as diet, alcohol consumption, smoking and physical activity, all of which may be risk factors for cancer death. Furthermore, there was no information on tumor grade, metastases or given treatment. Since we were not able to adjust analyses by these factors, confounding is possible. Smoking is a risk factor for both cardiac diseases and cancer death thus it could be a confounding factor increasing observed cancer mortality among antiarrhythmic drug users. We did not have information on frequency of health care contacts. This might have been greater among antiarrhythmic drug users and therefore leading to earlier detection of tumors, which might result in lower observed cancer mortality.

Our information on medication use is based on reimbursed drug purchases. We do not know for sure whether or not patients have actually used the drugs they have bought. Finally, the study population was originally recruited for a prostate cancer screening trial. The cohort included principally Caucasian men so there is no certainty whether the results can be generalized to women or other ethnic groups.

## Conclusion

We observed that antiarrhythmic drug use has neither general cancer protective effects nor a beneficial impact on any particular cancer type. In contrast, cancer mortality was increased among antiarrhythmic drug users compared to non-users, but the risk increase was likely non-causal as it was related to short-term use only and disappeared in long-term use. Our results do not support the hypothesis of digoxin’s anticancer effects or those of any other antiarrhythmic drug.

## Material and Methods

### Study cohort

We used the population of the Finnish Randomized Study of Screening for Prostate Cancer (FinRSPC), which is the largest component of the European Randomized Study of Screening for Prostate Cancer (ERSPC). The detailed trial protocol has been described previously^32^. In short, 80,458 men were recruited to the study during the years 1996–1999. Men were randomized to either the screening arm (31,866 men, prostate-specific antigen test at 4-year intervals) or control arm (48,278 men, no intervention, followed through national cancer registry). The follow-up continued until the end of 2015. Prevalent prostate cancer cases at baseline were excluded; no exclusions for other cancers were made.

The official causes of death in 1996–2015 were obtained from the death certificate registry of Statistics Finland. FinRSPC cause-of-death committee has previously found Statistics Finland to be a dependable source of data (kappa 0.95)^33^. The data included primary, immediate and contributory causes of death recorded as ICD-10 codes. For this study we collected information on deaths with lung (C34), colorectal (C18), pancreatic (C25), gastric (C16), liver (C22), renal (C64), non-Hodgkin lymphoma (C81), bladder (C67) or central nervous system cancer (C71 and C72) recorded as the primary cause of death. Prostate cancer deaths were included in overall cancer mortality. We have previously performed a separate analysis for the risk of prostate cancer death^17^.

Information on diagnoses recorded during in- and outpatient hospital contacts during 1996–2012 were obtained from the Care Register for Health Care (HILMO) of the National Institute for Health and Welfare. The data was used to calculate the CCI for the study participants. Additionally, we sought information on indications for antiarrhythmic drug use: heart failure (ICD-10 code I50) and cardiac arrhythmias (I47 and I49).

The study was approved by the Ethics Committee of the Pirkanmaa Health Care District, Finland (tracking number R10167) and the Committee confirmed that all research was performed in accordance with relevant guidelines. Informed consent was obtained from all participants in the screening arm of the study.

### Information on medication use

We collected data on antiarrhythmic drug purchases during 1995–2015 from the reimbursement database of the Social Insurance Institution of Finland (SII). SII is a governmental agency that provides reimbursements for physician-prescribed drug purchases to all Finnish citizens as part of a national health insurance. All reimbursed purchases are registered in the database that records the date, number of packages acquired, and number and dosage of the tablets for every purchase. This information allowed us to calculate the amount of the medication purchases for each drug on a yearly basis.

Antiarrhythmic drug purchases were identified with drug-specific ATC–codes. Drugs in clinical use during the study period were amiodarone, digoxin, disopyramide, etilefrine, flecainide, quinidine, mexiletine, procainamide, propafenone and sotalol. Additionally, we obtained information on use of statins, antidiabetic medication (oral glucose-lowering drugs and insulins), antihypertensive medication (beta-blockers, ACE-inhibitors/ATII receptor blockers, calcium-channel blockers, diuretics and other types of drugs, such as methyldopa and clonidine), aspirin and other NSAIDs, 5-alpha-reductase inhibitors and alpha-blockers.

### Statistical analysis

The baseline characteristics were compared between ever-users and never-users of antiarrhythmic drugs using the Chi-square test for categorical variables and the Mann-Whitney U test for continuous variables. The association between antiarrhythmic drug use and cancer mortality was estimated using the Cox proportional hazards regression. We estimated hazard ratios (HR) and their 95% confidence intervals (CIs) for cancer death overall and for deaths due to specific types of cancer by antiarrhythmic drug use. We analyzed mortality by overall antiarrhythmic drug use and separately by digoxin and sotalol use. The follow-up time was calculated from FinRSPC randomization to the date of death, emigration or the common closing date (December 31th 2015), whichever occurred first.

Antiarrhythmic drug use was analyzed as a time-dependent variable to minimize immortal time bias. Therefore, we updated the medication use status prospectively for every year of follow-up by annual medication purchases. If there was a recorded purchase at any point during a year, the man was regarded as a user. If medication purchases were stopped during the follow-up, the participant remained in the user category to minimize bias due to selective discontinuation of medication use in the terminal phase of cancer. Men without any purchases during the follow-up and all users before the first purchase were classified as non-users, which was used as the reference group in the main analyses. Age-adjusted and multivariable analyses (further adjustment for baseline cancer diagnosis and use of other drug groups: drugs used in management of benign prostatic hyperplasia, diabetes, hypercholesterolemia or hypertension, and aspirin and other NSAIDs) were conducted. Besides antiarrhythmic drugs, use of other drugs was included in the analyses as a time-independent variable.

We standardized amounts of antiarrhythmic drugs use by dividing the cumulative annual milligram amount of each drug with the standard Defined Daily Dose (DDD) published on the WHO website^34^. By adding together years with antiarrhythmic drug purchases, we were able to estimate cumulative duration of drug use. Intensity of drug use (DDDs/year) was calculated by dividing the cumulative annual amount with duration of medication use. We stratified men into tertiles by the variables mentioned above to estimate whether the amount or duration of drug use affects mortality.

Effect modification by age, baseline cancer, use of other drug groups and socioeconomic factors was evaluated in subgroup analyses stratified according to these variables. The statistical significance of each effect modifier was evaluated by adding an interaction term between antiarrhythmic drug use and the background variable into the multivariable-adjusted Cox regression model.

We evaluated the long-term effects of antiarrhythmic drug use in lag-time analyses, in which the time-dependent status of antiarrhythmic drug use was lagged forward 3–5 years in follow-up time. These analyses were carried out to minimize confounding by indication, as especially digoxin is commonly used in management of potentially lethal congestive heart failure. In addition, we performed competing risk regression analyses with non-cancer deaths as the competing risk. These analyses were conducted according to the method reported by Fine and Gray^35^.

The statistical tests were two-sided. P-values of 0.05 or less were considered statistically significant. IBM SPSS Statistics 23 (Chicago, IL, USA) software was used for data analyses.

## Electronic supplementary material

Supplementary tables S1 and S2
