# Supplementary material for: Cancer mortality does not differ by antiarrhythmic drug use: A population-based cohort of Finnish men
Source: Sci Rep. 2018 Jul 9;8:10308. doi: 10.1038/s41598-018-28541-4 (PMC6037774; doi:10.1038/s41598-018-28541-4)
Supplement: Supplementary file 1 — Supplementary tables S1 and S2 [file 41598_2018_28541_MOESM1_ESM.pdf]

Cancer mortality does not differ by antiarrhythmic drug use. A population-based cohort of Finnish men.

Kalle J. Kaapu, Lauri Rantaniemi, Kirsi Talala, Kimmo Taari, Teuvo L. J. Tammela, Anssi Auvinen, Teemu J. Murtola

Supplementary table 1. Antiarrhythmic drug use and individual cancer mortality in Finnish Randomized Study of Screening for Prostate Cancer.

	Gastric cancer death	Liver cancer death	Non-Hodgkin lymphoma death	Renal cancer death	Bladder cancer death	Central nervous system cancer death
	HR (95%CI)[a]	HR (95%CI)[a]	HR (95%CI)[a]	HR (95%CI)[a]	HR (95%CI)[a]	HR (95%CI)[a]
Antiarrhythmic drug use						
No use	Ref	Ref	Ref	Ref	Ref	Ref
Any use	0.95 (0.61-1.46)	0.96 (0.69-1.33)	1.99 (1.42-2.78)	1.26 (0.84-1.88)	1.57 (1.03-2.38)	1.25 (0.74-2.11)
Digoxin use						
No use	Ref	Ref	Ref	Ref	Ref	Ref
Any use	1.20 (0.75-1.93)	1.08 (0.75-1.55)	2.19 (1.50-3.21)	1.50 (0.96-2.35)	1.89 (1.19-3.00)	1.06 (0.54-2.09)
Sotalol use						
No use	Ref	Ref	Ref	Ref	Ref	Ref
Any use	0.80 (0.35-1.80)	1.32 (0.80-2.18)	0.77 (0.34-1.74)	0.82 (0.37-1.86)	0.68 (0.25-1.84)	0.96 (0.35-2.59)

[a] From Cox regression model adjusted for age and use of cholesterol-lowering, antidiabetic and antihypertensive drugs, aspirin and other NSAIDs, and 5alpha-reductase inhibitors and alpha-blockers

Supplementary table 2. Antiarrhythmic drug use and cancer mortality among men with no cancer diagnose at baseline in Finnish Randomized Study of Screening for Prostate Cancer.

	Overall cancer death [a]	Lung cancer death	Colorectal cancer death	Pancreatic cancer death
Antiarrhythmic drug use	HR (95%CI) [b]	HR (95%CI) [c]	HR (95%CI) [c]	HR (95%CI) [c]
No use	Ref	Ref	Ref	Ref
Any use	1.46 (1.36-1.57)	1.81 (1.59-2.07)	1.40 (1.10-1.78)	1.00 (0.77-1.31)
Digoxin use				
No use	Ref	Ref	Ref	Ref
Any use	1.63 (1.51-1.77)	2.20 (1.90-2.54)	1.62 (1.24-2.12)	1.07 (0.79-1.46)
Sotalol use				
No use	Ref	Ref	Ref	Ref
Any use	1.18 (1.04-1.35)	1.14 (0.87-1.48)	0.77 (0.45-1.30)	1.03 (0.65-1.62)

[a] Including lung, prostate, colorectal, pancreatic, gastric, liver, renal, non-Hodgkin lymphoma, bladder and central nervous system cancer

[b] From Cox regression model adjusted for age, screening trial arm and use of cholesterol-lowering, antidiabetic and antihypertensive drugs, aspirin and other NSAIDs, 5alpha-reductase inhibitors, alpha-blockers

[c] From Cox regression model adjusted for age and use of cholesterol-lowering, antidiabetic and antihypertensive drugs, aspirin and other NSAIDs, and 5alpha-reductase inhibitors and alpha-blockers
